# Overpassivization Revisited: Disentangling Syntax and Semantics in Causes of Errors

**DOI:** 10.1007/s10936-025-10192-y

**Published:** 2026-02-11

**Authors:** Chorong Kang, Eunjeong Oh

**Affiliations:** 1 Department of Linguistics, Seoul National University, 1, Gwanak-Ro, Gwanak-Gu, Seoul, 08826 Korea; 2 Department of English Education, Sangmyung University, 20, Hongjimun 2-Gil, Jongno-Gu, Seoul, 03016 Korea

**Keywords:** Overpassivization errors, Split intransitivity, Acquisition of prosody-syntax interface, Agentivity

## Abstract

English passives and unaccusatives share similarities in both syntactic derivation and semantic properties of the subject argument. However, similarities in syntactic derivation have primarily been discussed in the literature as a core trigger of prevalent overpassivization errors with unaccusative verbs by second language learners of English. In this study, we conducted two experiments to investigate which cue, either syntactic similarity or semantic similarity, is active for incorrectly applying passive frames to unaccusative verbs. The first experiment assessed whether learners represent unaccusatives as sharing a syntactic derivation with passives. Based on the assumption that stress patterns reflect syntactic derivations, participants judged acceptability of stress patterns of intransitives and passives. The results did not provide conclusive evidence that L2 learners treat unaccusatives and passives as sharing a common syntactic derivation. Even the five most native-like learners showed residual optionality, and their divergent performance on passive versus intransitive constructions indicates that highly advanced learners may still lack fully distinct syntactic representations for the two types of intransitives. In the second experiment, we investigated whether learners instead rely on thematic information. The results show that learners consistently rated unaccusative subjects as less agentive than unergative subjects, indicating that they recognize the thematic-role similarity between unaccusatives and passives. Taken together, the findings suggest that semantic similarity between passives and unaccusatives, rather than syntactic identity, drives learners’ overpassivization behavior.

## Introduction

English has two types of intransitives: one with unaccusative predicates (1a) and the other with unergative predicates (1b). One of the most intriguing phenomena in second language acquisition studies is that second language learners of English, across L1 backgrounds, tend to produce incorrect passive forms as in (1c) or reject correct intransitive forms with unaccusative verbs as in (1a) (Oshita, 1997; Yip, 1990; Zobl, 1989, among many others).

1. a. A boy appeared
  b. A boy smiled
  c. *A boy was appeared
  d. *A boy was smiled

Given that such overpassivization errors are rarely observed with unergative verbs (1d), a natural question to ask might be why unaccusative verbs are vulnerable to overpassivization errors. Over the past decades, the similarity in the underlying (syntactic) structure of passives and unaccusatives has been understood as the fundamental reason for overpassivization errors (Oshita, 1997; Yip, 1990, 1995; Zobl, 1989). The Unaccusative Hypothesis, proposed by Perlmutter (1978), posits that unaccusative verbs differ from unergative verbs in terms of the underlying position of the subject argument; the subject of unaccusative verbs is base-generated within VP like the subject of a passive predicate, while that of unergative verbs originates outside of VP like the subject of a transitive predicate. The syntactic view of overpassivization errors has been developed based on the assumption that both a passive subject and an unaccusative subject undergo local A-movement from their base-generated position within VP to SpecTP in L2 learners’ grammar. Zobl (1989) argues that the “passivized” unaccusative structure serves as a “mark” of the local A-movement, which L2 learners might apply to the argument base-generated within VP. This syntax-based account views L2 learners’ overpassivization errors as evidence supporting the idea that the subject of unaccusative verbs is correctly projected within VP in L2 learners’ grammars. A corpus study by Oshita (1997) also reports similar phenomena: L2 learners are more likely to (i) avoid the canonical subject NP-V word order, and (ii) accept V- subject NP sequences, with or without expletives, when the verb is an unaccusative verb, compared to when it is an unergative verb. He interprets this pattern as an indication that L2 acquirers hesitate to treat unaccusative subjects as canonical transitive subjects or unergative subjects. Based on the observation, Oshita suggests the “unaccusative trap” hypothesis, proposing that overpassivization errors emerge during the process of learning the syntactic structure of intransitives. While different syntactic accounts may vary in detail, they all share the same intuition regarding unaccusative predicates: L2 learners may correctly move the internal argument of unaccusatives to SpecTP, but incorrectly extend the morphological passive marking (be + -ed) to these verbs, overgeneralizing the passive morphology to a structure that already involves subject movement for independent reasons.

Nevertheless, recent studies have reported variation in error rates among unaccusative verbs, indicating that syntactic explanations alone may be insufficient and that additional factors must be considered. Various non-syntactic factors have been proposed, including the influence of L1 morphology (No and Taegoo 2006; Kondo, 2005), discourse pragmatics (Ju, 2000), subject animacy (Okamura & Shirahata, 2024; Pae et al., 2014), the presence of an alternating transitive counterpart (Hwang, 2006), and the interaction between more than one source (Lee & Choi, 2011). Although previous studies have discussed additional non-syntactic factors influencing overpassivization, those studies typically maintain the view that syntactic derivation is the primary source of the asymmetry between unaccusatives and unergatives.

The goal of this paper is to re-examine the commonly held view that the syntactic similarity between passives and unaccusatives underlies overpassivization errors. We question whether this syntactic explanation truly holds for L1-Korean learners, whose L1 provides little evidence for a structural distinction between unergatives and unaccusatives, as will be discussed in the next section. We address this question through two experiments.

Experiment 1 examines learners’ sensitivity to prosodic cues that reflect the underlying syntactic structure of intransitives (as will be discussed in Sect. "Syntactic derivation and prosody"). If learners show native-like prosodic differentiation, it would suggest that they may possess syntactic knowledge of the unaccusative–unergative contrast. Experiment 2 investigates whether thematic or agentivity-based cues can better account for learners’ patterns. As is well known, the subjects of unaccusatives and passives share the same thematic role, either a theme or a patient, devoid of agentivity (Perlmutter, 1978; Burzio, 1986; Baker, 1988; Levin & Malka, 1995). Thus, there is a possibility that overpassivization errors stem from a mere “analogy” between passives and unaccusatives, grounded in the thematic role of subjects, without necessarily considering syntactic derivations. If this holds true, the presence of the passive morpheme with unaccusative verbs would be independent of the L2 learners’ syntactic knowledge. Instead, L2 learners might simply use the passive morpheme to spell out the lack of agent when the subject functions as a theme or a patient.

Unlike previous studies, the present study does not measure overpassivization frequency itself. Instead, it aims to determine whether L2 learners construe the two intransitive classes as syntactically similar to passives (through structural derivation) or as semantically similar (through agentivity or patienthood). We believe this provides new insight into the underlying grammatical knowledge that shapes learners’ errors.

The remainder of the paper is organized as follows. Sect. "Syntactic and Semantic Properties of the Two Types of Intransitives" presents the theoretical background for the two experiments: Sect. "Syntactic derivation and prosody" discusses how prosodic patterns relate to syntactic structure, and Sect. "Thematic roles of the subject of intransitives" examines the relevant semantic properties of intransitives. Sect. "Experiments" reports the two experiments and the results. Sect. "General Discussion" provides the general discussion and conclusion.

## Syntactic and Semantic Properties of the Two Types of Intransitives

### Syntactic Derivation and Prosody

It is reported in the literature that unaccusatives and unergatives exhibit different prosodic patterns in English (Schmerling, 1976; Zubizarreta & Vergnaud, 2006; Kratzer & Selkirk, 2007). When the verb is an unergative verb, as in (2a), sentence stress is more likely to fall on the verb. Conversely, when the verb is an unaccusative verb, it is more natural for sentence stress to be placed on the subject, as in (2b) (capital letters indicating sentence stress).

(2) a. a student WALKED
  b. a STUDENT arrived

This empirical judgment has received confirmation through several experimental studies. Nava and Zubizarreta (2009) conducted a production test wherein the experimenter and the participant engaged in a scripted dialogue in a Question–Answer format. They observed that when native English speakers produce a sentence with an unaccusative verb in a wide-focus context (i.e. where the sentence conveys all-new information), sentence stress typically falls on the subject 97 percent of the time (3). In contrast, when native English speakers produce a sentence with an unergative verb in a wide-focus context, sentence stress is distributed between the subject (49%) and the verb (51%), as depicted in (4).

(3) Q: What was that crashing sound? (Nava & Zubizarreta, 2009: (51))
  A. A GLASS broke
(4) Q: What was the noise in the waiting room? (Nava & Zubizarreta, 2009: (53))
  A. A PATIENT sneezed. A patient SNEEZED

Irwin (2011) also investigated English native speakers producing sentences with unergative and unaccusative verbs. The results revealed a significant difference in pitch and duration between the subject noun and the verb in unaccusative sentences compared to unergative sentences. Specifically, the subject in unaccusative sentences exhibited significantly greater prominence compared to the verb bearing high pitch and longer duration. Such difference was not observed in unergative sentences. Regarding intensity, the relative difference between the subject and the verb in unaccusatives and unergatives did not show a significant difference. However, the data indicated that the relative difference in the mean intensity between the subject and the verb in unaccusative sentences was higher than in unergative sentences. Therefore, while intensity did not show a significant difference, the intensity results were in line with the findings in pitch and duration.

Production tests conducted by Nava and Zubizarreta (2009) and Irwin (2011) show the same pattern that is in line with the long-standing empirical observations about the prosodic characteristics of the two types of intransitives. What is interesting to the current discussion is that such a stress pattern is closely associated with syntactic derivations. Two approaches to the syntax-prosody interface have been suggested to account for these prosodic patterns. The traditional view suggests that sentence stress falls on the last stress-bearing element in VP (Cinque, 1993; Legate, 2003; Zubizarreta, 1998). In contrast, the phase-based view, named by Irwin (2011), posits that sentence stress is assigned to the highest element in the spell-out domain (Irwin, 2011; Kahnemuyipour, 2004; Kratzer & Selkirk, 2007). In the following, we illustrate how each approach accounts for the prosodic differences between the two types of intransitives.

Since Cinque (1993), building on the work of Chomsky and Halle (1968), the Nuclear Stress Rule (NSR) has been assumed to assign primary sentence stress to the final stress-bearing word within VP. An example of the traditional view is illustrated in Legate (2003).^1^ With the assumption that unaccusatives have a VP phase, the subject-accent pattern can be accounted for as follows: In (5a), the subject *a window* was originally generated as the last element inside VP. VP is the first spell-out domain and contains a copy of *a window* within VP. Thus, the NSR applies onto VP and the sentence stress falls on the last element *a window* (capital letters indicating sentence stress). Later, the subject *a window* moves to its surface position, SpecTP, and it is overtly pronounced there with the sentence stress as shown in (5b). This illustrates how the subject of unaccusatives is realized with the sentence stress. On the other hand, the subject of unergatives is base-generated outside of VP. Thus, only the verb is found inside the VP, as shown in (6). When the NSR applies onto VP, the sentence stress falls on the sole element within VP, the verb *ran*. This explains the observed verb-accent pattern in unergatives.

(5) a. [_VP_ broke a WINDOW]]. By NSR
  b. [_TP_ A WINDOW [_VP_ broke a WINDOW]]. Subject movement to SpecTP
(6) [_TP_ A child [_VP_ RAN]. By NSR

This traditional approach predicts that sentences with unaccusative verbs are biased to the subject-stress pattern, while sentences with unergative verbs are biased to the verb-accent pattern. However, as observed in previous experimental studies, the biased prosodic pattern in unergatives is relatively weaker than in unaccusatives. In other words, many instances of unergatives are realized with the subject-accent pattern as well. Thus, this analysis does not straightforwardly account for the prosodic variability within unergatives. However, we do not argue that this analysis is incompatible with the empirical data. It might explain the prosodic variability among unergatives if additional assumptions are introduced. What we hope to highlight is that different prosodic patterns are associated with distinct syntactic properties of the underlying structure: since the subject of unaccusatives is base-generated within VP, the sentence stress falls on the subject noun, whereas the subject of unergatives is base-generated outside of VP, so the sentence stress falls on the verb (in many cases).

The second approach is the phase-based approach. Kahnemuyipour (2004, 2009) proposes that sentence stress falls on the highest constituent in a spelled-out domain (see Kratzer & Selkirk, 2007 for some variation of the same approach). Building on Chomsky (2000, 2001), Kahnemuyipour assumes that unergative and transitive *v*Ps are phases, while unaccusative and passive *v*Ps are not phases. Since the only phase head is C due to the lack of *v*P phase in unaccusatives and passives, as demonstrated in (7a), the subject is the structurally highest phrase in the spell-out domain, resulting in the subject-stress pattern. On the other hand, since unergatives have the *v*P phase as in (7b), the highest constituent in the first spell-out domain is the verb. Similar to the traditional view, this analysis needs further elaboration to account for the prosodic variability within unergatives.
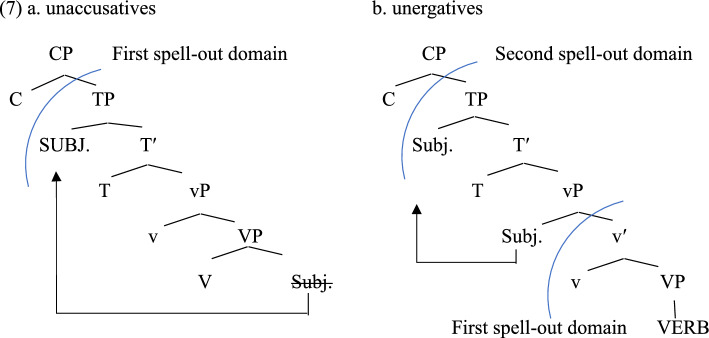


We have explored two different approaches to account for the prosodic variability between the two types of intransitives. Even though assumptions and the exact mechanisms of each approach vary, both accounts converge on the same idea: First, sentence stress assignment is linked to syntactic structural properties; Second, the difference in the base position of the subject gives rise to prosodic variation. In a narrow-focus context or a topic-comment context, stress patterns are influenced by semantic or pragmatic reasons. However, in a wide-focus context where none of the constituents in the sentence are semantically highlighted or given, the stress pattern is determined based on the syntactic derivation. This implies that the stress pattern native English speakers produce reveals the syntactic structure they employ in producing the sentence.

Given the discussion thus far, we assume that if L2 learners exhibit a preference for different stress patterns depending on the types of intransitives, similar to the patterns observed in native English speakers, then this would suggest that they discern the syntactic distinction between unaccusatives and unergatives. In the current work, we experimentally investigate whether Korean L2 learners exhibit a preference for different stress patterns in the two different types of intransitives. We are well aware that L2 learners’ responses on prosody do not directly reveal their knowledge about syntactic structures. This type of experiment inherently assumes that participants require not only knowledge of syntactic derivation but also an understanding of syntax–prosody mapping, such as the Nuclear Stress Rule (NSR), in order to determine whether a given prosodic pattern is appropriate. In other words, this task is designed to prompt participants to utilize their knowledge at the syntax-prosody interface. According to the Interface Hypothesis (Sorace & Filiaci, 2006), certain interfaces pose greater challenges for L2 learners to acquire compared to other interfaces or non-interface syntax (i.e., narrow syntax). The debate continues which interface is more challenging to acquire (White, 2011). Therefore, if Korean L2 English learners fail to distinguish between unaccusatives and unergatives in terms of prosody, it suggests that either L2 learners have not fully acquired the derivational differences between the two intransitive structures or they have not fully acquired the syntax-prosody interface despite successfully acquiring each derivation within the narrow syntactic domain.

Nevertheless, we maintain that the experimental method is worth pursuing for the following reasons. First, no direct evidence has been observed in the error patterns of L1-Korean L2 English learners. In his corpus study, Oshita (1997) reports that Spanish and Italian L2 English speakers tend to produce a V–subject NP word order with unaccusative verbs. In contrast, Korean and Japanese L2 English speakers rarely exhibit such a pattern (with only a single case observed from a Japanese speaker). Oshita highlights that L2 speakers’ error patterns vary based on their L1 background. Given that Spanish and Italian feature an unmarked postverbal subject with unaccusative verbs, L2 speakers with Spanish or Italian L1 background may be more likely to be sensitive to the base position of the subject, hinted at by the surface structure of their native languages. Despite differences in the set of unaccusative verbs between English and Spanish/Italian, Spanish/Italian L2 speakers might take advantage of information inferred from their first language. If this is the case, one could suspect that Spanish/Italian learners of English “know” the base position of the unaccusative subject even in their non-native language, namely English. Then, what does it say to us that the V-subject NP word order is rarely observed in the production of L2 English speakers with Korean as their first language? Korean is a free word order language due to scrambling, so the V-subject NP order is licit although it is a marked one. Crucially, there is no distinction between unaccusatives and unergatives regarding the availability of the postverbal subject structure in Korean. The V-subject NP order is purely pragmatically driven, independent of the verb types. As shown in the examples in (8) and (9), both unergatives and unaccusatives can occur in either subject NP-V order or V-subject NP order, with no discernible difference in naturalness between the two verb types.

(8) a. Emma-ka wus-ess-ta (Unergative)
      mom-Nom laugh-past-decl
  b. Wus-ess-ta emma-ka
      laugh-past-decl mom-Nom
      ‘Mom laughed.’
(9) a. Emma-ka salaci-ess-ta (Unaccusative)
      mom-Nom disappear-past-decl
  b. Salaci-ess-ta emma-ka
      disappear-past-decl mom-Nom
      ‘Mom disappeared.’

The distribution of the V-subject NP order in Korean is unaffected by the fact that the verb is an unergative or an unaccusative verb. This stands in contrast to Spanish and Italian. In these languages, postverbal subjects are significantly more likely to appear with unaccusative verbs than unergative verbs (Hertel, 2003). Thus, there exists an asymmetry between unaccusative and unergative verbs concerning the distribution of the postverbal subject structure. In contrast, Korean speakers face challenges in obtaining information about the base position of unaccusative subject from their native languages. Oshita’s observation suggests that there may be insufficient evidence to argue that Korean learners know the base position of unaccusative subjects in English.^2^ In addition, we have not found any corpus study documenting these specific errors among Korean learners of English.^3^Therefore, the projection of the subject of unaccusative verbs within the VP in Korean learners’ grammar remains questionable.

Second, we incorporated passive constructions into our experiment. Many English textbooks used by Korean students present passive constructions as sentences involving the promotion of an object to the subject position by adding the passive morpheme (be + Past Participle). This description explicitly teaches learners the syntactic derivation of passives. Thus, if learners do not behave like English native speakers in passive constructions, it will indicate that Korean speaking English learners have difficulties in the syntax-prosody interface.

On the other hand, if learners behave like English native speakers in passive constructions but not in intransitives, the source of difficulty might lie in the syntactic derivation of intransitives.

Furthermore, there is an additional benefit to this experimental method. If the results show that L2 learners do not pattern like native English speakers, although we are not sure whether L2 learners have fully acquired the syntactic derivations of intransitive constructions, the results could serve as supporting evidence for the “hard-to-acquire” nature of the syntax-prosody interface. Therefore, in our first experiment, we tested Korean L2 English speakers’ judgments on prosodically manipulated English intransitives.

In this study, we employed a comprehension test rather than a production task. Previous researches have shown that overpassivization errors appear not only in production but also in sentence-judgment tasks, suggesting that these errors stem from learners’ underlying grammatical representations rather than production-specific limitations. For this reason, we consider both production tasks and comprehension tests to be informative. Since comprehension is generally assumed to precede production in acquisition, and since the syntax–prosody interface is relevant in both modalities, a perception-based method is also theoretically justified. To bridge the methodological gap between earlier production-based studies for English native speakers and our perception-focused approach, we tested English native speakers as a control group. Their responses confirmed that perceptual sensitivity to the stress patterns of intransitives aligns with the production patterns reported in previous studies. Consequently, we adopted a comprehension test with the expectation that it would more sensitively detect learners’ underlying grammatical knowledge in this hard-to-acquire domain than a production task would.

We also believe that such a new perspective is worthy in the situation where no syntactic tests can probe into the syntactic derivation independent to other intertwining factors. For example, resultative and causative alternation have been considered to be a diagnostic for unaccusativity (Levin & Malka, 1995; Lee, 2004; Baker et al. 2013; Baker, 2015). However, it is reported that some, but not all, unaccusatives pass the diagnostic. For example, non-alternating unaccusative verbs like *arrive*, as in (10b), does not show causative-inchoative alternation.

(10) a. The chocolate melted/ The chef melted the chocolate
  b. The letter arrived/ *The postman arrived the letter

Several studies have suggested potential factors influencing the verbs that allow resultatives/causative alternation (Levin & Malka, 1995; Ramchand, 2008, among many others). These studies all assume that syntactic underlying structure alone cannot delineate the condition on resultatives or causative alternation. Instead, more complex semantic factors must be considered for this distinction. Thus, testing syntactic constraints requires a deeper understanding of semantic aspects, such as telicity and semantic initiator, extending beyond the scope of the syntactic knowledge we aim to investigate. Thus, testing syntactic constraints might evaluate L2 learners’ sensitivity to differences in telicity or causativity. Previous studies have explored Korean L2 learners’ understanding of the syntactic properties of unergatives and unaccusatives using syntactic diagnostics.^4^ For example, Park (2006) conducted a series of experiments assessing the acceptability of resultatives and pseudopassives with unergative and unaccusative verbs. The results indicated that, regardless of proficiency level, Korean learners did not consistently align their judgments with those of native English speakers. Even though the results indicated that Korean speakers did not accurately respond to resultatives and pseudopassives, it would be premature to conclude that Korean learners do not know the different underlying structures of the two types of intransitives, as other semantic factors are intertwined in these tests. Unfortunately, other syntactic diagnostics suggested in the literature are only applicable to a subset of unaccusatives as wel1 although overpassivization errors are observed across unaccusative verbs, making it insufficient to generalize the results of tests on syntactic constraints to all unaccusatives (Baker, 2015).^5^

Therefore, instead of examining L2 learners’ knowledge of syntactic constraints related to split intransitives, we opted to test L2 learners’ judgments on prosody, allowing us to minimize the impact of complex syntactic and semantic factors on participants’ judgments. Importantly, although previous research has examined Korean learners’ knowledge of unaccusatives using various syntactic diagnostics, no prior work has investigated how this knowledge relates to prosodic awareness or stress-pattern interpretation. Because the syntax–prosody interface has not been explored as a diagnostic domain in the acquisition of split intransitivity, our study provides the first attempt to connect L2 learners’ unaccusative representations with their prosodic sensitivity.

To summarize, the preceding discussion has shown that prosodic realization is closely tied to syntactic derivation: differences in sentence stress patterns between unergatives and unaccusatives arise from how their subjects are structurally positioned. However, prosodic prominence and thematic interpretation belong to different representational domains. The prosody–syntax interface concerns how structural information is mapped onto phonological form, whereas thematic roles are defined in the event structure based on the semantic relation between the predicate and the argument. In the following section, we turn to the semantic dimension of the problem, asking whether learners treat the thematic role of the unaccusative subject in the same way as that of the passive subject, and whether overpassivization errors arise from an analogy such that a non-agentive subject is associated with the passive morpheme.

### Thematic Roles of the Subject of Intransitives

As we will show in this section, the patientlike properties of the subjects of unaccusatives are another potential factor that might lead L2 English learners to make overpassivization errors. The subjects of unaccusatives are entities that are affected by the event described by the predicate, as in (11a).

(11) a. A glass fell (on the floor)
  b. A patient sneezed

Such a patientlike reading of the subject possibly reminds L2 learners of passive sentences that also contain a patientlike subject. Thus, it is possible that L2 learners add passive morphemes when the subject of a sentence is a patient or a theme by analogy with passive sentences. This heuristic behavior does not necessarily require L2 learners to possess syntactic knowledge about the underlying structure of unaccusatives. Although syntactic base position and thematic roles are closely connected in theoretical syntax, L2 learners can potentially rely on these cues differently in overpassivization contexts. Without considering the syntactic operation, namely A-movement, L2 English learners can apply such an analogy in the way that a non-agent, patientlike subject ought to be followed by a passive predicate. In contrast, the subjects of unergatives are entities that cause or perform the event described by the predicate, as in (11b). Therefore, the heuristic rule by the analogy with passives cannot be applied to unergatives, resulting in a lower overpassivization error rate.

The effects of certain semantic properties on overpassivization errors have been attested in the literature. Pae et al. (2014) investigated how the animacy of the subject affects the rate of acceptance of incorrect passive sentences. They found that Korean learners of English make more overpassivization errors when the subject of unaccusatives is an abstract noun than when it is a human or a concrete noun. However, there was no significant difference in error rates between human subjects and nonhuman concrete subjects. This study suggests that a specific thematic property of the subject may induce more errors, but it remains unclear whether agentivity or animacy is the locus of the error-prone thematic property.

Ju (2000) found that L2 English learners, whose native language is Chinese, were more likely to accept passivized unaccusatives when an external cause or agent was cognitively imaginable compared to scenarios where an external cause was not conceivable. The results show that English learners accept a verb in a particular syntactic form not only based on the lexicosemantics of the verb but also due to cognitive factors such as the source of the cause. As subjects in externally caused events can be readily interpreted as patients, the results indicate that the patienthood of the subject in intransitives could play a role in overpassivization errors.

Jo (2018) conducted a corpus study revealing that Korean EFL learners are more likely to use incorrect passive sentences with unaccusative verbs when the verb appears with an inanimate subject as opposed to an animate subject. This result supports the idea that subject animacy plays a role in the tendency to make overpassivization errors. The findings from the corpus studies are worthy of further consideration given that animacy does not strictly correspond to agentivity (or causativity) and the characteristic thematic property of the subjects of unaccusatives is patienthood (i.e., lack of agentivity) rather than inanimate nature. Animacy is “an inherent property of concepts” (Rosenbach, 2002:42), so a noun bears [± animate] features independent of the context of a sentence. On the other hand, agentivity is assigned in a context. It has been pointed out in the literature that animacy and agentivity must be dissociated. Givón (1993) suggests that agentivity is evaluated based on volition, control, and responsibility rather than animacy. Schlesinger (1989) argues that feature *Cause* is the core property of an agent presenting examples independent of animacy (e.g., *The dishwasher cleaned the dishes,* Schlesinger (1989:193)). Likewise, a theme cannot be defined by [-animate] as well. It is not difficult to find an animate theme (e.g., *The ball hit the man*). Given the characteristic properties of an agent, we assume that the characteristic properties of a theme can be defined as the lack of causality, volitionality, or responsibility. Thus, further research is needed to explain how animacy, not being a characteristic thematic property of the subject of unaccusatives, can affect overpassivization errors. It could be argued that Korean learners are more likely to use passive morphologies when the verb appears with an inanimate subject than an animate subject because inanimate nouns are more easily interpreted as themes than animate nouns. This perspective suggests that animacy itself may not directly affect overpassivization errors but rather serves as a by-product of the L2 speakers’ analogy between passives and unaccusatives based on the patienthood property of the subject. If this holds true, we could understand the discrepancy in the overpassivization error rate between unaccusatives and unergatives as a consequence of Korean learners’ interpretation of the thematic roles of the subject. Such a view predicts that Korean learners are more likely to interpret the subject of unaccusatives as non-agent compared to that of unergatives, a prediction tested in our second experiment.

To the best of our knowledge, there’s limited research distinguishing between structural and semantic cues as potential sources of errors in overpassivization. We seek to address this gap by applying a double dissociation technique to investigate this issue. Specifically, we can examine overpassivization errors in two scenarios: when predicates do not take a theme-subject, and the subject is base-generated within VP, or when predicates take a theme-subject but the subject is base-generated outside of VP. If overpassivization errors are observed in the first case, it suggests that these errors are triggered by the syntactic cue. On the other hand, if overpassivization errors are observed in the second case, they can be considered triggered by the semantic cue.

Unfortunately, both cases are challenging to test empirically. According to the Uniformity of Theta Assignment Hypothesis (UTAH) by Baker (1988), an NP base-generated within VP cannot take an agent theta role but can take a patient or a theme role. This makes it logically difficult to find examples for the first case. For the second case, if we follow the lexical approach to middle constructions (Ackema & Schoorlemmer, 1995 or Fagan, 1988), looking at overpassivization errors with middle constructions could be a candidate because the subject is base-generated outside of VP but it bears a theme (or patient) thematic role.^6^ However, since almost every verb in middle constructions has a passive counterpart, passivization is grammatically possible. Therefore, distinguishing the syntactic cue from the semantic cue by examining overpassivization errors seems to be unavailable.

Given that a direct empirical dissociation between syntactic and semantic cues is not feasible, an alternative approach is required to determine which source of information actually underlies learners’ overpassivization behavior. For this reason, we investigate their sensitivity to prosodic and thematic properties as a way to infer the underlying grammatical representations that shape overpassivization. In Experiment 1, we tested how Korean L2 English learners represent the syntactic structures of English intransitives by assessing their sensitivity to prosodic patterns. In Experiment 2, we examine how L2 learners represent the thematic role of subjects. Together, these two experiments aim to disentangle syntactic and semantic contributions to overpassivization.

## Experiments

In this section, we present the two experiments. In the first experiment, we tested how Korean learners of English judge the prosodic patterns of English intransitives. This experiment is designed to indirectly investigate their representation of the syntactic structures of intransitives. Since prosody is not intertwined with characteristic semantic properties that might distinguish unaccusatives from unergatives (e.g., telicity), the experiment is expected to investigate English learners’ syntactic knowledge of English intransitives independently of any compounding semantic factors such as telicity or thematic relations. If learners prefer the subject-accent pattern over the verb-accent pattern only in unaccusatives, but not in unergatives, as observed in native English speakers’ data, it could provide compelling evidence supporting the idea that L2 learners understand the difference in syntactic derivation between the two types of intransitives and how it is manifested in prosody.

To maintain consistency with the first experiment, we conducted a comprehension test for the second experiment as well.^7^ In the second experiment, on the other hand, we tested how Korean learners understand the thematic roles of subjects in the two types of intransitives. It also provides evidence supporting the idea that agentivity, independent of animacy, is the locus of the discrepancy between unergatives and unaccusatives in the overpassivization error rate.

### Experiment 1

#### Participants

Ninety-six adult native Korean speakers participated in the experiment. They were all undergraduate students of Sangmyung University and Korea University. None of the participants reported being bilingual in Korean and another language. Participants were categorized into three English proficiency levels using the Michigan English Test (MET): 22 advanced, 54 intermediate, and 20 beginners. All participants were living in Korea at the time of testing. Additionally, we recruited 26 native English speakers for a native control group.

#### Materials

We manipulated two factors: (i) predicate types (unaccusatives, unergatives, and passives) and (ii) prosody (sentence stress on the subject noun or on the verb), resulting in a total of six conditions, as shown in Table 1.

**Table 1:** Experiment design

		Predicate types		
		Unaccusatives	Unergatives	Passives
Stress assignment	Subject	a BABYcame	a BABY swam	a BABY was found
	Verb	a baby CAME	a baby SWAM	a baby was FOUND

**3 × 2 Design** *predicate types* (unaccusatives vs. unergatives vs. passives) X *stress assignment* (subject vs. verb)

A total of 24 verbs (8 for each predicate type) were used in the target sentences, as listed in (12).

(12) Verbs used in stimuli.
  (i) Unaccusative verbs: come, die, fall, appear, disappear, arrive, rise, vanish.
  (ii) Unergative verbs: swim, cry, run, agree, whisper, smile, run, crawl.
  (iii) Passive verbs: find, raise, hit, attack, change, love, hurt, choose.

The target items exclusively feature non-alternating unaccusative verbs, excluding alternating unaccusative verbs. Previous studies have shown that Korean learners of English are more likely to make overpassivization errors with alternating unaccusatives than non-alternating unaccusatives (Hwang, 2006, Kim, 2004, Kim, 2010, No & Taegoo, 2006, Oh, 2014). According to those studies, Korean learners of English are reluctant to use the intransitive word order with alternating unaccusative verbs because these verbs appear in the three varieties (transitives, passives, and intransitives) in their input and the passive varieties with a patient subject in their input interferes with the production or processing of intransitives with those verbs. Considering that unergative verbs appear only in the intransitive form in their input, to maintain maximal input uniformity between the two verb conditions, we excluded alternating unaccusative verbs from the target items. The stimuli include non-alternating verbs that most frequently lead to overpassivization errors based on the corpus study by Song and Oh (2021).

To ensure animacy control, each target sentence featured a human subject noun. Thus, unaccusative verbs that are not semantically plausible with a human subject (e.g., *happen*, *occur*) were excluded, despite their frequent appearance in incorrect passive forms. A native English speaker confirmed the semantic and pragmatic coherence of each target sentence. Unergative verbs were selected to closely match the vowels of unaccusative verbs (e.g., *die-cry*, *fall-run*).

Passive verbs were included in the stimuli for comparison with unaccusatives. Both passives and unaccusatives share the same underlying structure and syntactic derivation, including local A-movement. Thus, these two types of constructions are expected to exhibit the same prosodic pattern, where the prominent sentential accent falls on the subject. This prediction is borne out (Kahnemuyipour, 2004; Okazaki, 1995). If Korean L2 English learners use passive morphemes with unaccusative verbs due to the similarity in syntactic derivation, it is expected that they will prefer assigning the same prosodic pattern to both sentence types.

Each participant listened to a single subject item (e.g., *baby* in the above example) paired with only one type of verb and in one stress assignment condition, preventing inter-item interference. Every participant heard all 24 target verbs, with half in the subject-accent condition and the other half in the verb-accent condition. A trained male native English speaker recorded each target sentence, along with filler sentences, in a sound-attenuated booth. Items were pseudo-randomized within each list.

Each target sentence, consisting of an article *a*, a subject noun, and a verb, formed a single intonational phrase without pauses between words. Unaccusative and unergative verbs were paired, aiming to match the number of syllables within each pair. However, due to the limitation of the list of available unaccusative verbs which frequently appear in overpassivization errors, it was impossible to perfectly match the segments making up the stressed syllable within a pair.

One might wonder whether the intrinsic pitch, intensity, or duration differences between different segments might affect the perception of the stress pattern. To avoid such a potential concern, the audio stimuli were analyzed to confirm the manipulation of prosody. For all test sentences, mean/maximum pitch, duration, and intensity of the nucleus of the stressed syllable for subjects and verbs were measured. Results, summarized in Figs. 5, 6, 7 and 8 in the Appendix, showed consistent patterns. Regardless of the type of verbs, mean pitch of subjects was higher than verbs in the subject-accent condition, and vice versa in the verb-accent condition, with no notable differences between verb types. The results of the F0 maximum pattern together with the results of the pitch mean. For every verb condition, in the subject-accent condition, mean of the maximum pitch of the subjects is higher than that of the verb, while in the verb-accent condition, the mean of the maximum pitch of the verbs is higher than that of the subjects. Likewise, the intensity analysis supports the validity of the manipulation of prosody. Mean intensity of the stressed syllable of the subjects is higher than that of the verbs in the subject-accent condition across the verb conditions. Lastly, duration analyses also pattern together.^8^ The acoustic analyses of the auditory targets support that the manipulation of prosody is well in line with the purpose of our experimental design. In the verb-accent condition, verbs were significantly more prominent than subjects in terms of higher pitch, greater intensity, and longer duration, and vice versa in the subject-accent condition (except duration). Crucially, no noticeable differences between verb types were observed.

A total of 32 filler items, comprising 16 transitive sentences and 16 *wh*-interrogatives, were randomly interspersed among the target stimuli. All audio stimuli, including targets and fillers, were presented with a wide-focus inducing context. In each trial, participants read one of four variations of “what happened” (e.g., *John is talking about what happened yesterday.*) displayed on the monitor 500 ms before they listened to the audio stimuli.

#### Procedure

Participants were tested individually in a sound-attenuated room, facing a computer screen. They read a description of the experimental procedure from a printed sheet, complemented by verbal instructions from an experimenter. Their task involved listening to the audio stimuli and judging the naturalness of prosody in each sentence. Participants were explicitly instructed that all sentences were grammatically correct, and their focus should be on evaluating prosody. Using a scale from 1 to 5 (where 1 is the worst and 5 is the best), they were to judge how natural the prosody sounded within the given context.^9^

Given that Korean learners of English often face challenges in listening comprehension, visual stimuli accompanied the audio stimuli, presenting target and filler sentences in written form. The order of presentation for the two types of stimuli was carefully considered. Studies on silent reading have revealed that people activate phonological features, including prosody while processing visually presented linguistic targets (Bader, 1998; Hwang & Schafer, 2009). Due to the implicit prosody during the silent reading, if the visual stimuli were presented before or simultaneously with the audio stimuli, participants might activate their own prosody, so the implicit prosody could affect their decision on the audio stimuli. To avoid such a situation, we presented each audio stimulus twice: once 500 ms before and once 500 ms after the corresponding visual stimulus. The initial audio presentation aimed to preemptively block potential implicit prosody triggered by the visual stimulus, while the subsequent presentation provided participants with a second chance to make judgments, especially considering potential difficulties arising from their listening comprehension challenges.

Before the test, participants went through a practice session. Experimental items were presented in a fixed-random order, ensuring that no more than two consecutive items belonged to the same experimental condition.

#### Predictions

If Korean learners of English have learned that the two types of intransitives have distinct syntactic derivations, and if they understand that syntactic derivation feeds prosody, they would likely judge the subject-accent condition better than the verb-accent condition in the unaccusative-verb condition and the passive condition. However, such a preference would not be observed in the unergative-verb condition. On the contrary, if they do not understand the derivational differences between the two types of intransitives or the prosodic realization of the syntactic derivations, the expected systematic preference would not be observed. In a scenario where learners have acquired knowledge of how prosody is intertwined with syntactic derivation but do not fully grasp the syntactic derivation of different types of intransitive verbs, they would probably judge the subject-accent condition more favorably than the verb-accent condition in passive constructions but not in unaccusative constructions.

#### Results

**Fig. 1:**
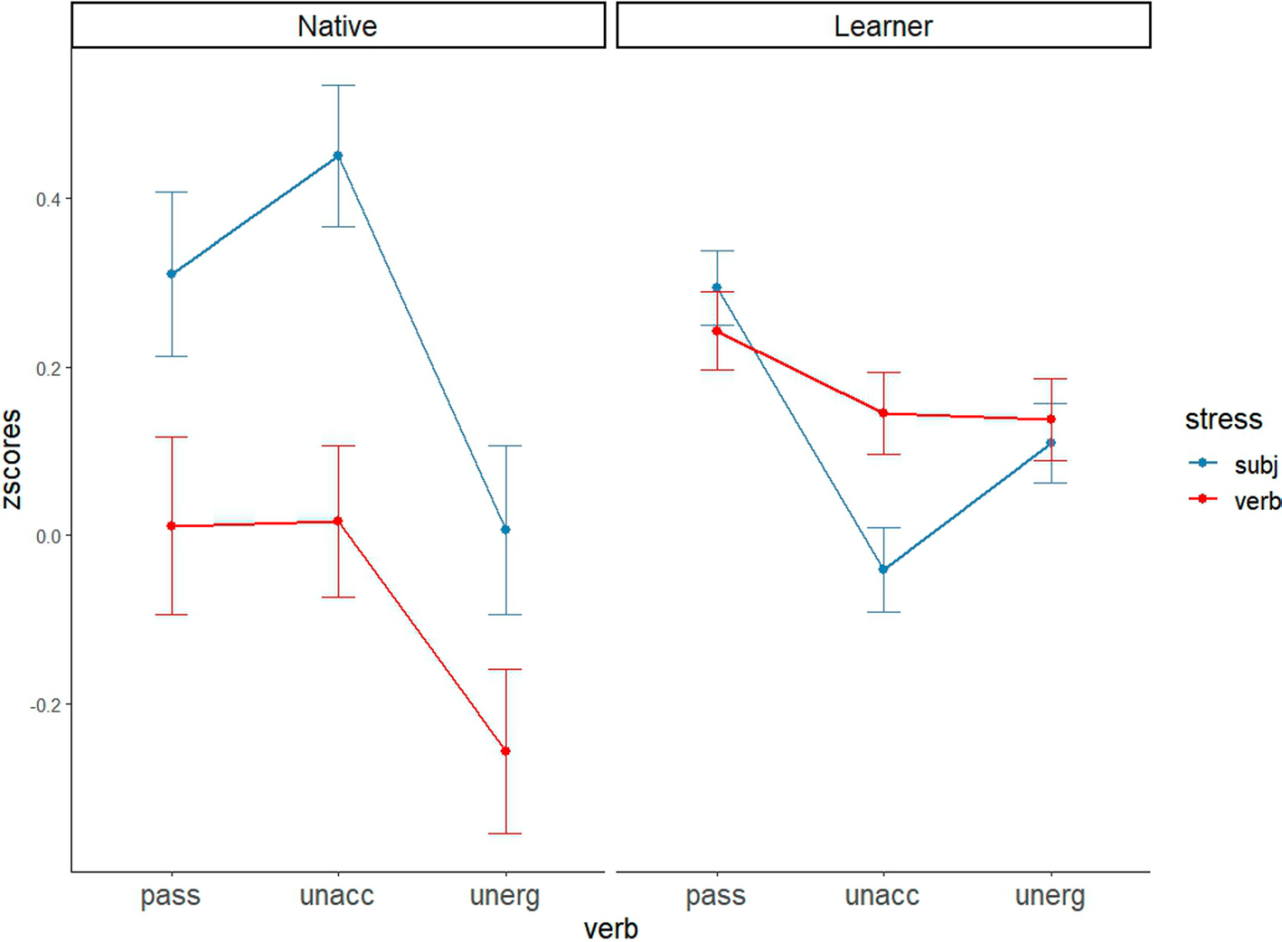
Overall results

For each condition, the dependent measure was the scores participants gave. Participants’ responses were transformed into z-scores for further analysis. Figure 1 shows the mean z-scored responses, along with standard error of the mean (SEM) error bars, for each experimental condition within each participant group.

To better understand the relationship between stress patterns and verb types, linear mixed effects models were constructed in R (R Core TeAM, 2020, version 4.0.2) using the lme4 package (Bates et al., 2015) and lmerTest (Kuznetsova et al., 2017). Models were constructed for each participant group (native speakers, advanced learners, intermediate learners, and beginners). P-values for the main effects were obtained through model comparison (comparison between the full model with the effect in question and a reduced model without the effect in question, (Ackerman, 2018)). The model incorporated a maximal random effect structure, or reduced maximal random effect structure if the former failed to converge (Barr et al., 2013).

In the full model, fixed effect predictors included verb-type and accent-type, along with the interaction term. Random intercepts for both subject and item were incorporated and a by-subject random slope was included for the effects of verb types and accent types. For the reduced model without the effect of the verb, accent-type was entered as the sole fixed effect predictor. Similarly, for the reduced model without the effect of the accent, verb-type was the only fixed effect predictor. In the reduced model without interactions, both verb-type and accent-type were included as fixed effects, excluding the interaction term.

Responses from native English speakers are congruent with previous studies. A significant main effect of stress-type was observed, indicating that native English speakers consistently prefer the subject accent condition over the verb accent condition, regardless of Verb type (χ^2^(1) = 7.62, *p* < 0.01). However, the gap in the acceptability of each accent condition differs by verb type. Using a Bonferroni correction, we conducted a pairwise t-test. Native English speakers significantly prefer the subject accent condition over the verb accent condition when the verb is an unaccusative verb (*p* < 0.01) or a passive verb (*p* < 0.05). In contrast, there is no significant difference in preference between the two stress patterns when the verb is an unergative verb. The results are consistent with the notion that passives and unaccusatives share a similar syntactic derivation, leading to a similar prosodic pattern.

Further pairwise comparisons highlight that responses to passives tend to align more closely with those to unaccusatives than with unergatives. Specifically, the acceptability of unaccusatives and passives with sentential stress on the subject is significantly higher than that of unergatives with the same stress pattern (unaccusative x subject_accented vs. unergative x subject_accented, *p* < 0.05; passive x subject_accented vs. unergative x subject_accented, *p* < 0.05). Notably, there is no significant difference between unaccusative verbs and passive verbs in the subject-accent condition. This indicates that native English speakers are more inclined to accept the subject-accent condition with both unaccusative and passive verbs compared to unergative verbs.

**Fig. 2:**
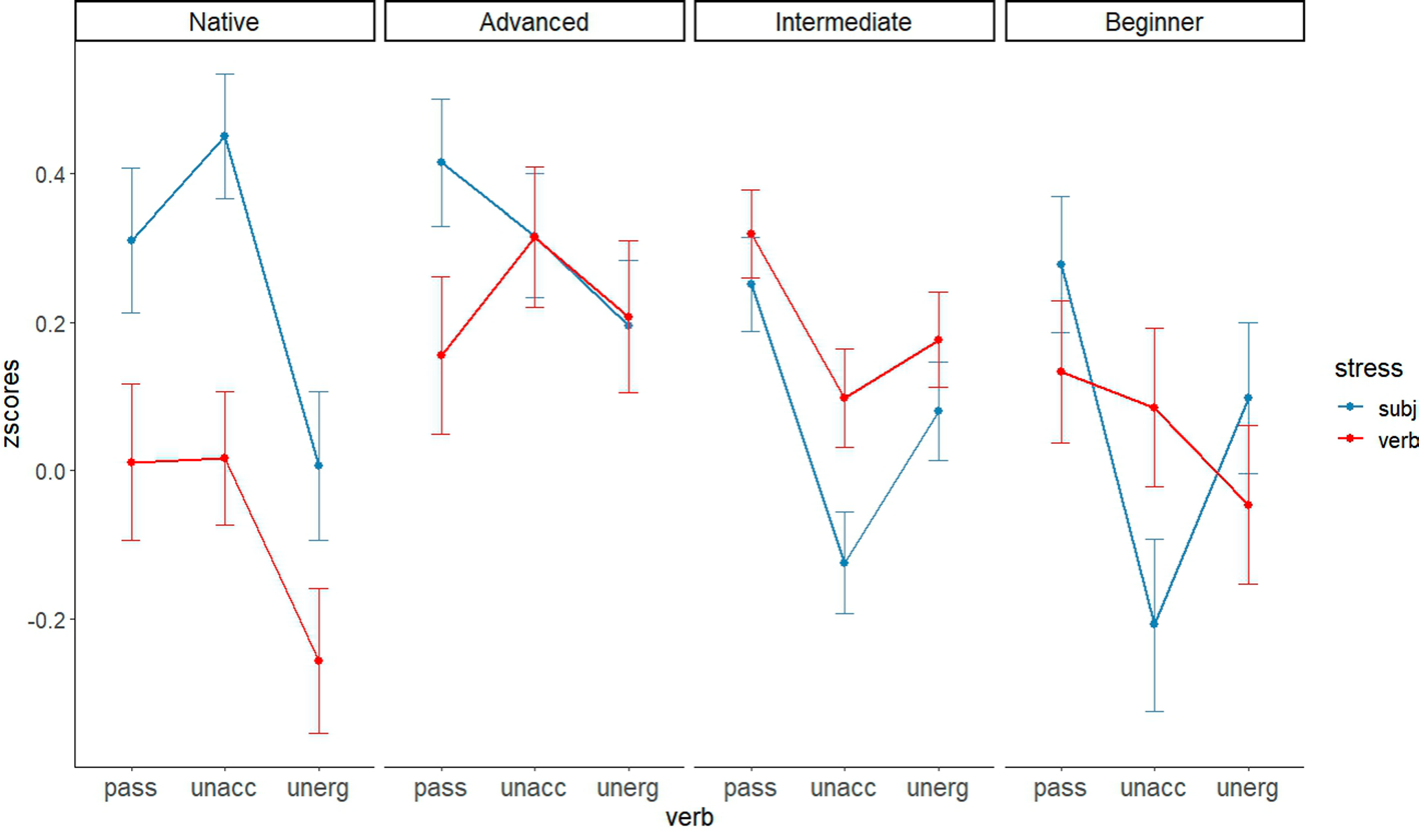
Mean z-scores of each condition by participants’ level in Experiment 1

To better understand Korean learners’ responses, instead of looking at the learners’ results in Fig. 1 altogether, we opted to examine the results based on learners’ proficiency levels, as illustrated in Fig. 2.

Learners across all proficiency levels did not show the same behavior as native English speakers. Beginners exhibited a subject-accent preference in both the unergative and passive conditions. However, within each verb type, the difference between the subject-accent and verb-accent conditions did not reach significance. Thus, it seems premature to conclude that beginners prefer the subject-accent condition to the verb-accent condition with passives or unergatives.

The intermediate group also did not show a preference for the subject-accent condition with passives or unaccusatives. Across all verb conditions, the acceptability of the two accent conditions did not differ significantly.

**Fig. 3:**
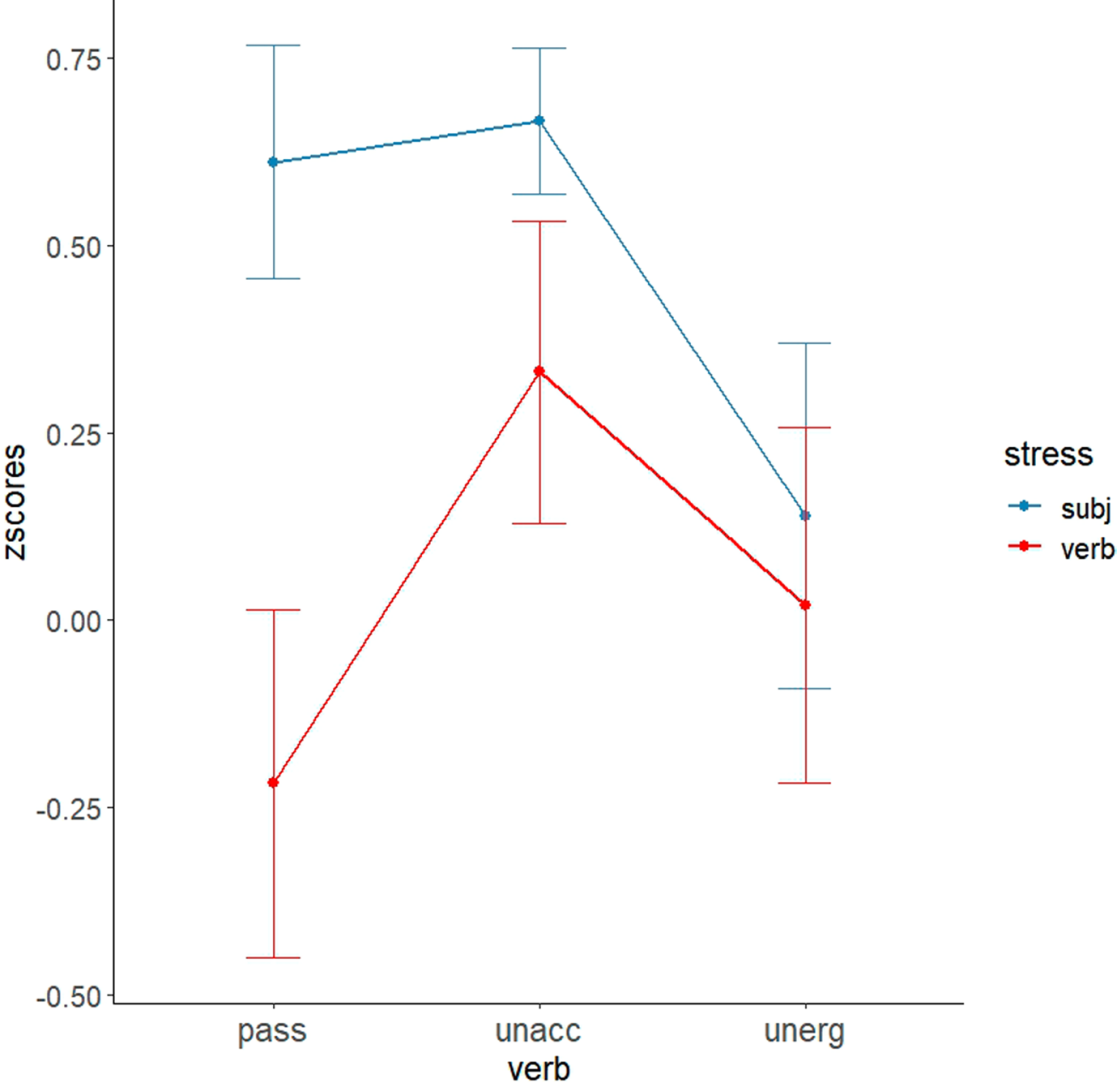
Mean z-scores of five highly advanced L2 learners

Finally, even in the advanced group, no significant difference between the two accent conditions was observed for any verb type. As shown in Fig. 2, the advanced group did not distinguish between the two types of intransitives in terms of prosody. However, we found that advanced speakers marginally preferred the subject-accent condition to the verb-accent condition in the passive condition (*p* = .058). To see whether highly advanced learners show a residual optionality as predicted by the Interface Hypothesis, we extracted out the five participants’ responses, who earned more than 27 points out of 30 points in the proficiency tests. The z-scores are plotted in Fig. 3. Although the data points are small (5 participants × 24 items = 120 trials), the results seem to be substantially different from either the native English speakers’ responses or the overall advanced learners’ responses. The highly advanced learners judged the subject-accent condition to be significantly more acceptable than the verb-accent condition in the passive context. A pairwise t-test revealed a significant difference between the two accent conditions (*p* < .01). This trend appears somehow similar to the native English speakers’ responses but slightly different from the overall advanced learners’ responses. However, they still highly accepted the verb accent condition when the unaccusative verb was used, and as a result, we did not observe a significant difference between the two stress conditions in the unaccusative condition, contrary to the results observed in native speakers’ responses. Given the small number of participants and data points, further research is warranted to delve into this issue. Nevertheless, the results suggest that highly advanced learners show a residual optionality in learning the syntax-prosody interface, showing both target-like responses and non-target-like responses.

One noticeable observation is that, despite the challenging nature of the prosody-syntax interface, highly advanced learners exhibit a native speaker-like pattern in the passive verb condition (i.e., a significant difference between the two prosody conditions), but not in the unaccusative verb condition (i.e., the lack of a significant difference between the two prosody conditions). Upon further analysis of the five highly advanced learners’ responses, it became evident that all of them noticeably preferred the subject accent condition in the passive constructions. Interestingly, among these advanced learners, only three participants' responses aligned with those of native English speakers' responses in the two intransitive conditions. Specifically, these three participants preferred the subject accent condition to the verb accent condition with unaccusative verbs, but showed no preference for the stress pattern in the unergative condition, similar to native speakers’ responses. These individual analyses indicate that highly advanced learners’ reactions to prosodic patterns differ between passives and unaccusatives.^10^

#### Discussion

Before discussing the main implication of the current experiment, we need to explain why native English speakers in the current experiment prefer the subject-accent condition over the verb-accent condition not only in unaccusatives but also in unergatives. We suspect the reason for the overall preference of the subject-accent condition lies in the thetic interpretation of the target sentences. Sasse (1987) argues that stress pattern varies by the way an event is described. If a sentence presents a simple event (thetic interpretation), sentence stress is more likely to be on the subject. On the other hand, if a sentence has a topic-comment structure indicating that the predicate presents a statement about a subject, the sentence stress is more likely to be on the verb. All the target sentences used in the current experiment tend to be thetic statements since the verbs are not statives but eventives, which are more likely to have a thetic judgment. We believe that the biased thetic interpretation in a wide-focus context drives the overall subject-accent preference.^11^ Yet, the difference in the relative preference of the subject-accent condition over the verb-accent condition between unaccusatives and unergatives is remarkable given that the two types of intransitives are all biased to have a thetic judgment.

Unfortunately, we did not find evidence supporting the hypothesis that Korean learners of English are sensitive to prosodic variation between the two types of intransitives. Furthermore, only the highly advanced learners patterned like native speakers with passives. Previous studies on the acquisition of English NSR have highlighted the challenges faced by L2 learners in acquiring prosodic constraints at the syntax–prosody interface (DeKeyser, 2007; Nava & Zubizarretta, 2009; Lu & Se-Eun, 2016). Almost all of these studies examined L2 speakers whose L1 also employs the NSR (e.g., Spanish, Chinese) and attributed difficulty in acquiring English NSR to L1 prosodic transfer. The results of the current study show that difficulty in the syntax–prosody interface domain is observed even when learners’ L1 does not apply the NSR at all. In other words, the difficulty is not restricted to languages that share a similar prosodic system, but emerges across typologically different systems as well.

The overall lack of a subject-accent preference in passive constructions among Korean learners suggests that they have not yet acquired how to apply the NSR to specific syntactic structures, with the exception of the highly advanced learners. Consequently, our results remain inconclusive as to whether learners understand the syntactic distinction between the two types of intransitives or insufficient acquisition at the syntax–prosody interface may obscure syntactic knowledge. However, a marginal subject-accent preference was observed in the advanced group in the passive condition (*p* = .058), and the responses from the five highly advanced learners showed a significant preference for the subject accent in the condition. This suggests that sufficiently advanced learners may appropriately apply the NSR to English sentences once the relevant syntactic derivation has been acquired, as appears to be the case for passives. Such native-like performance indicates that prosodic judgments are informed by syntactic knowledge. If this interpretation is correct, then the non-native-like responses in the intransitive conditions suggest that even (highly) advanced learners may not have fully acquired the syntactic derivation or underlying structure of intransitives. Testing a larger sample of highly advanced learners in future research is needed to provide a clearer picture.

The current experiment employed a task that does not rely on semantic properties. Our goal was to investigate whether L2 learners’ overuse of the passive morpheme with unaccusative verbs could be attributed to perceived syntactic similarities between unaccusatives and passives. Although the results do not provide conclusive support for this hypothesis, the performance of the highly advanced learners suggests that the syntactic derivation of unaccusatives may not be fully aligned with that of passives, even among learners at this level of proficiency. Thus, these results imply that a more effective approach to explaining biased overpassivization errors may require moving beyond syntactic factors to consider alternative cues that L2 speakers might rely on. One promising candidate is thematic role assignment, motivating the design of Experiment 2 to test this hypothesis.

### Experiment 2

#### Participants

Ninety-three adult native Korean speakers, who did not participate in the first experiment, participated in experiment 2, comprising 21 advanced, 52 intermediate, and 20 beginner participants. All were undergraduate students of Sangmyung University and Korea University.

None of the participants reported being bilingual in Korean and another language. The Michigan English Test (MET) was conducted to assess participants’ English proficiency. The participants were living in Korea at the time of testing. Additionally, 26 native English speakers were recruited for a native control group.

#### Materials

The verbs used in Experiment 1 were also used in the target sentences of Experiment 2, consisting of 8 unaccusative verbs, 8 unergative verbs, and 8 passive verbs.^12^ Additionally, 8 transitive verbs were included to provide participants with examples of typical agentive subjects. Thus, target sentences included typical agent-like subjects (in transitives) and typical patient-like subjects (in passives). This allowed participants to establish a range of scores for agentivity based on the two types of syntactically-cued subject arguments: transitive subjects on the one end and passive subjects on the other end. Examples of each verb type are given in (10).

(10)  a. *Transitive*: A kid baked a pie
   b. *Passive*: A baby was found
   c. *Unaccusative*: A student died
   d. *Unergative*: A child cried

The purpose of Experiment 2 is to determine whether Korean L2 English speakers distinguish the subjects of the two types of intransitives based on agentivity. In general, Korean L2 English speakers typically learn unaccusatives and unergatives as belonging to the same verb class, namely intransitive verbs, without being explicitly taught the unaccusative-unergative distinction. As participants progress through the experiment, they might recognize three sentence types: intransitives, transitives, and passives. The subjects of transitives were expected to be evaluated as the most agentive-like subjects due to the presence of the theme entity which is realized as the object. The subjects of passives were considered the least agentive-like subjects due to the overtly realized passive morphemes. Since there are only three sentence types, participants could potentially adopt a strategy-based decision without considering the meaning of the sentence. For example, they might assign a score of 5 (the most agentive-like) to the subjects of transitive sentences, 1 (the least agent-like) to passives, and a middle score to intransitives. To prevent such a situation, 8 copular sentences were added to introduce more complexity to the types of target sentences. Nevertheless, such a design is very conservative to our hypothesis: Korean L2 English learners recognize the difference in agentivity between unaccusatives and unergatives. If the prediction holds true even in the experimental design that disguises unaccusatives and unergatives as the same sentence type, the results would provide stronger support for our hypothesis. All subjects were human-denoting nominals to ensure that animacy-related variation did not affect the results. Each participant viewed 32 verbs, each with a different human subject, and no more than two consecutive verbs belonged to the same verb type.

#### Procedure

Participants were tested individually in a sound-attenuated room, seated in front of a computer screen, following the same setup as in Experiment 1. Each participant viewed a sentence on the computer screen and judged the volitionality of the subject of the sentence by assigning a score from 1 to 5. Drawing on Givón (1993), participants were asked to consider both the “volitionality” and the “ability to control the event.” A fixation point appeared at the center of the screen, and 500 ms later, the target sentence replaced the fixation point. Participants pressed a designated key on the keyboard to provide a score, after which the fixation point for the next trial appeared. Clear instructions were given, prompting participants to assign a score of 5 (highest) if they believed the subject of the sentence had high volitionality and significant control over the event described by the verb, or a score of 1 (lowest) if the subject demonstrated minimal volitionality or lacked control over the event. Intermediate scores (2, 3, and 4) were used to express varying degrees of volitionality observed in each subject.

#### Predictions

If Korean learners of English distinguish the subjects of the two types of intransitives based on agentivity, recognizing that the thematic role of unergative subjects is similar to that of transitive subjects, whereas the role of unaccusative subjects is similar to that of passive subjects, they should assign higher scores to unergative/transitive subjects than to unaccusative/passive subjects. However, if they do not make this agentive distinction and instead treat the thematic roles of unergative and unaccusative subjects as equally (non-)agentive, then their scores may not show a systematic difference between the two verb types.

#### Results

**Fig. 4:**
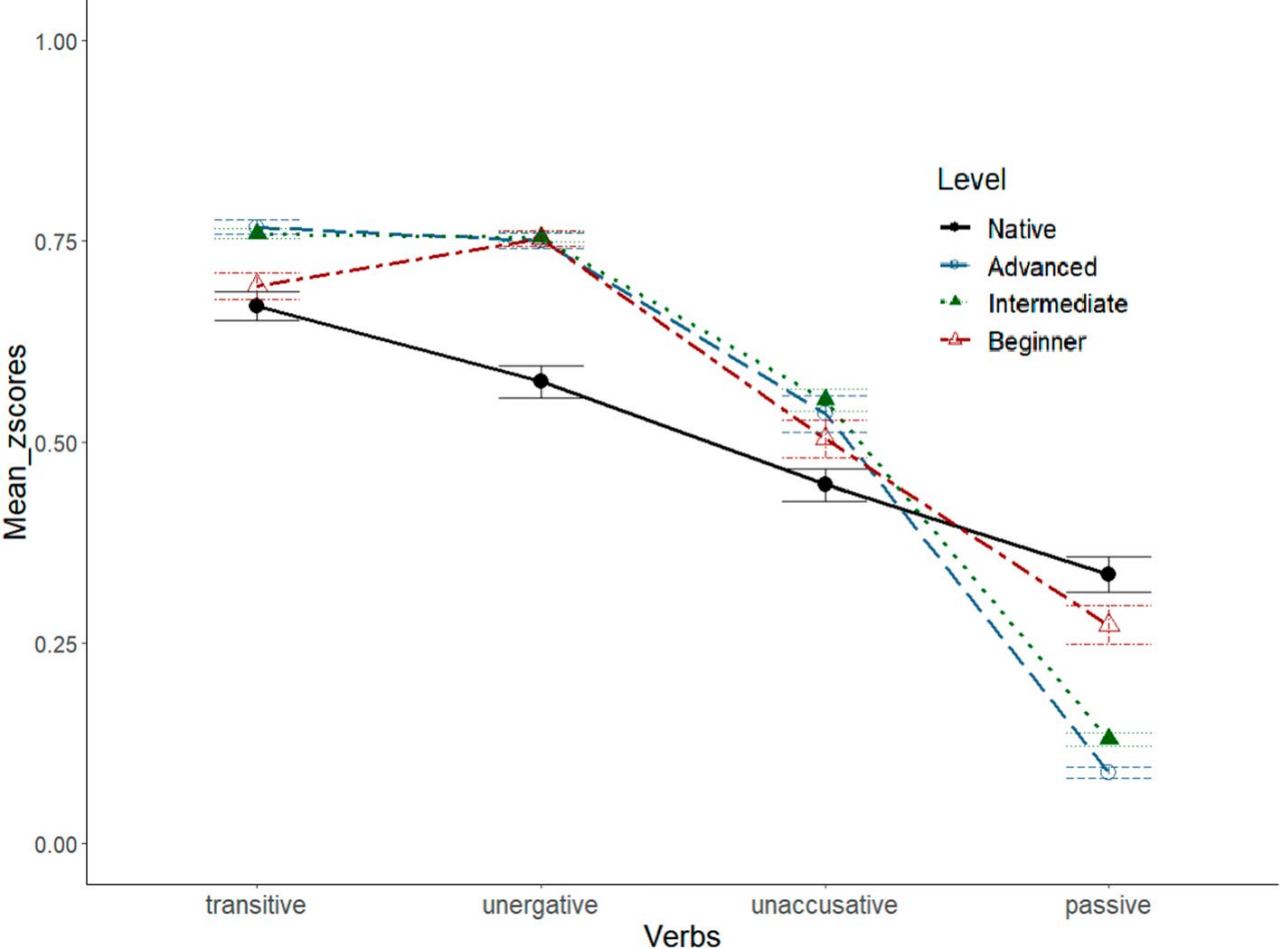
EXP2. Mean z-scores of each VerbType condition by participants’ level

**Fig. 5:**
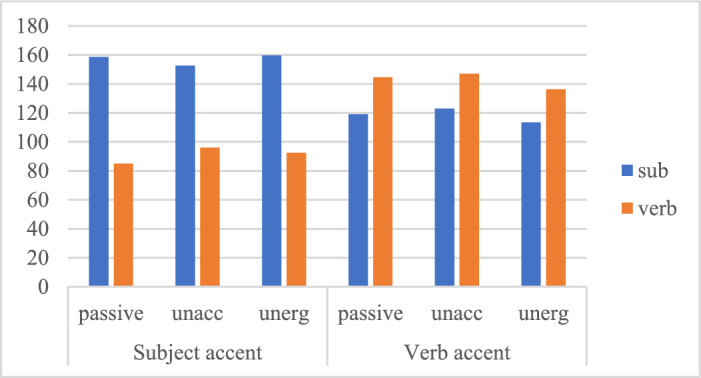
Mean F0 of target audio stimuli

**Fig. 6:**
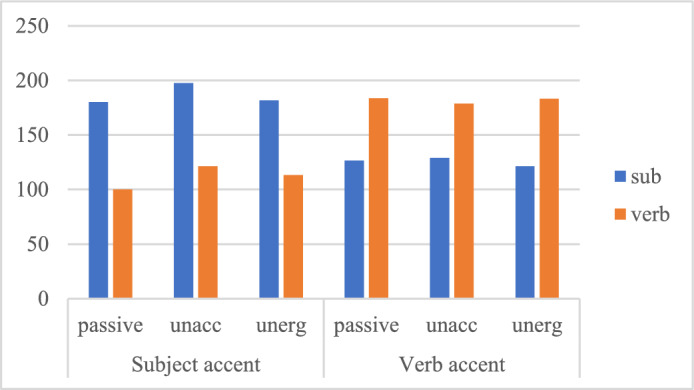
F0 maximum of target audio stimuli

**Fig. 7:**
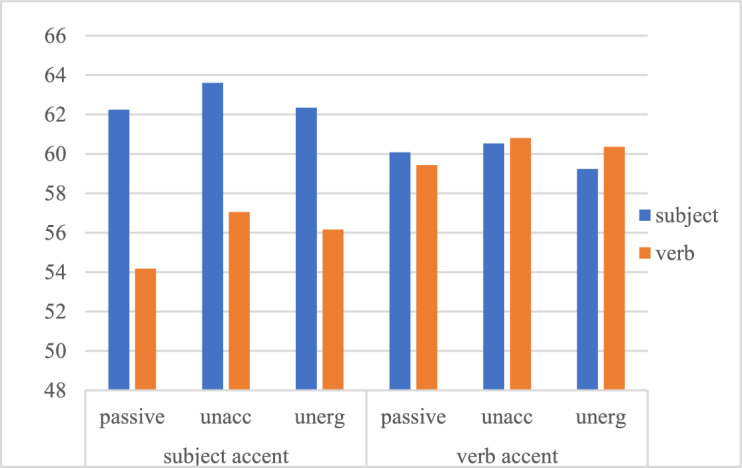
Intensity (mean dB) of target audio stimuli

**Fig. 8:**
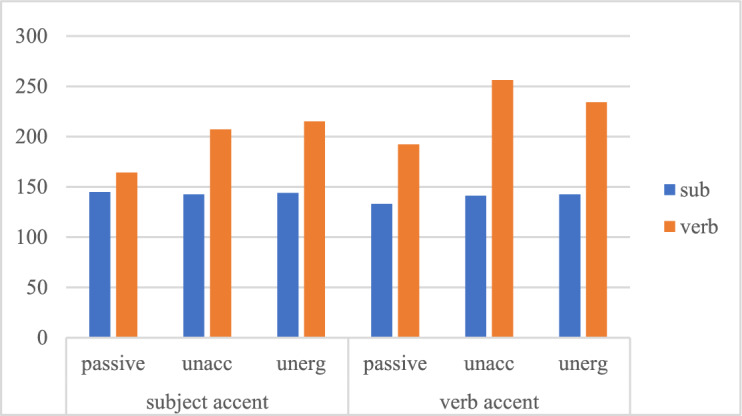
Duration of target audio stimuli

As in Experiment 1, responses were z-score transformed by participant, and mixed effects models were constructed in R. For fixed effects, we included verb-type and participant status (native vs. learners) and/or verb-type and proficiency (advanced, intermediate, beginner) in the model. The models comprised random intercepts for both subject and item. The mean z-scores and SEM error bars are summarized in Fig. 4.

A significant main effect of verb type was observed (χ^2^(6) = 331.72, *p* < 0.01), implying that participants’ responses significantly vary by verbs. Our primary interest is in examining how Korean speaking English learners recognize the agentivity of the subject of the two types of intransitives. For pairwise comparison, we used treatment coding, setting the reference level as desired for each comparison. Interestingly, L2 learners, regardless of their proficiency level, judged that the subjects of unergatives bear significantly higher agentivity (in terms of volitionality and being a controller of the event) than those of unaccusatives (β = 0.22, SE = 0.06, |t|= 3.41, *p* < 0.05). There was no significant main effect of proficiency level among learners: χ^2^(4) = 6.53, *p* = 0.16). Such a discrepancy between the two verb conditions was also observed in native speakers’ responses (β = 0.12, SE = 0.05, |t|= 2.78, *p* < 0.05). The results show that learners distinguish the agentivity of the subjects of the two types of intransitives, similar to how native English speakers do. Animacy does not play a role at all because all the subjects in the target sentences were human subjects. In addition, the two types of intransitives share the same surface word order, giving a (potentially misleading) impression that they share the same syntactic structure. Nevertheless, learners detect the semantic difference between the subjects of the two types of intransitives.

Even though learners judge the agentivity of unaccusative subjects significantly lower than that of unergative subjects, they also assigned significantly higher scores to the subjects of unaccusatives than to the subjects of passives (β = -0.39, SE = 0.06, |t|= 6.04, *p* < 0.01). However, there is no significant difference (but a marginal difference) between unaccusatives and passives in native speakers’ responses (β = -0.11, SE = 0.05, |t|= 2.12, *p* = 0.08). Based on the results, we suspect that native speakers regard the subjects of unaccusatives as a patient/theme entity, similar to passive subjects, while Korean learners consider unaccusative subjects as neither a theme/patient nor an agent but somewhere in-between.

In addition, learners show bipolar responses. L2 speakers judged the agentivity of transitives significantly higher than native English speakers (β = -0.08, SE = 0.02, |t|= 4.74, *p* < 0.01), while they judged the agentivity of passives significantly lower than native speakers (β = 0.18, SE = 0.02, |t|= 11.2, *p* < 0.01). L2 learners regard the agentivity of the subjects of unergatives as similar to transitive subjects, which is significantly higher than the judgment by native speakers (β = 0.18, SE = 0.02, |t|= 10.89, *p* < 0.01).

#### Discussion

Experiment 2 was designed to investigate the hypothesis that Korean learners of English distinguish between the two types of intransitives based on the thematic role of the subject. If they subconsciously recognize that subjects of passives and unaccusatives share similar thematic roles, characterized by being less agent-like, they might aim to represent this semantic similarity in their syntactic forms. This could explain why Korean learners of English make overpassivization errors with unaccusative verbs. Furthermore, if they have a sense that unergative subjects do not share this non-agent-like property, they would be less likely to use passive morphemes with unergative verbs, resulting in fewer overpassivization errors with this verb type.

The results partially support this idea. Korean learners of English judged that the subject of unaccusatives has less agentivity than the subject of unergatives, which patterns together with the well-known observation that most overpassivization errors occur when the verb is an unaccusative rather than an unergative. They may apply the passive morpheme to express the semantic relation between the event (described by the predicate) and the subject. In contrast, they judged the subject of an unergative sentence as having higher agentivity, which may account for the previous observations that Korean learners rarely produce incorrect passive sentences with unergative verbs.

We are aware that defining “agentivity” using semantic properties is challenging. In our experiment, participants were asked to evaluate the “volitionality” and “ability to control the event.” These properties are not categorical but scalar, meaning the same sentence (e.g., *A kid fell*) can be perceived as less volitional (e.g., because of strong wind) or more volitional (e.g., attempting to win a game) depending on the context. We did not provide any specific context; participants likely evaluated the volitionality of subjects by imagining various situations. From the results, we can infer that L2 learners can envision situations in which the subject performs a volitional action more readily when the following predicate is a transitive/unergative verb compared to when it is an unaccusative verb and least likely when it is a passive predicate. Thus, it appears that the less a subject is considered volitional, the more likely overpassivization occurs.^13^

Such an idea seems to be supported by one more interesting correlation between the list of verbs that Korean learners of English make many overpassivization errors with and the list of verbs whose subject is evaluated as having less agentivity. According to Jo (2018, pp52), *die* and *disappear* are the verbs that show a higher overpassivization error rate while *arrive* and *rise* are the verbs that show a lower overpassivization error rate. In Experiment 2, we used 8 unaccusative verbs: *die, fall, vanish, disappear, rise, appear, arrive, come*. The mean z-scores of each verb vary between 0.30–0.71. Interestingly, those verbs that are reported to elicit more overpassivization errors match with those verbs whose subject is judged less agentive-like (*die, disappear, appear*). Likewise, those verbs that are reported to elicit fewer overpassivization errors match with those verbs whose subject is judged more agentive-like (*arrive, rise*). Given the lack of a clear correspondence between these two factors, further investigation of this issue is warranted. Our emphasis here is on the impression that this observation conveys, namely, that thematic similarity between passives and unaccusatives, rather than syntactic similarities, appears to be a promising alternative in explaining the core reason for the biased overpassivization errors.

If the thematic relation between the subject and the predicate is indeed the fundamental cause of many overpassivization errors with unaccusative verbs, it becomes puzzling to observe a significant difference in the responses of learners between passives and unaccusatives. One possible explanation is a task-related effect: learners might have perceived unaccusatives presented in the form of intransitive sentences, as comparable to passive sentences that inherently feature patientlike subjects. Due to the disparity in surface syntactic structure, learners could have developed a response pattern, assigning the lowest score to passives and a higher score to the subjects of intransitives if they sounded less agentive. Another potential explanation is that agentivity is by nature scalar so that unaccusative subjects bear less agentivity than passive subjects even when they still bear much less agentivity compared to transitive/unergative subjectsin learner’s grammar. Sorace (2000) proposes an unaccusativity hierarchy that categorizes intransitive verbs along a continuum from the most prototypical unaccusative verbs to the most prototypical unergatives, taking into account differences in telicity of verbs and the agentivity of subjects. According to this hierarchy, even among unaccusatives, the agentivity of subjects can vary. Hence, the degree of agentivity might differ between the subjects of passive sentences and unaccusative sentences, despite both being labeled as “theme” or “patient” in terms of thematic roles. Considering that passive sentences involve a hidden or covert agent, participants might perceive the subject of passive sentences as bearing less agentivity. Without definitive evidence, we cannot conclusively determine whether this is solely a task effect or an indication that Korean learners do not view the subject of unaccusatives as a typical patient. This issue remains open for exploration in future studies.

## General Discussion

This work presents two experiments to disentangle the syntactic and semantic cues that may contribute to overpassivization errors among Korean L2 English speakers. The goal is to investigate the primary cause leading learners of English to make more overpassivization errors with unaccusatives than with unergatives. Assuming the UTAH is on the right track, the similarity in the underlying structure of unaccusatives and passives is inevitably associated with the similarity in the theta role borne by the subject. However, from a learnability perspective, L2 speakers can independently apply these two cues. In other words, it needs to be clarified whether L2 learners apply either one of the syntactic/semantic cues or both. Experiment 1 used a semantics-independent task to test Korean learners’ syntactic knowledge. If distinctive responses to the two types of intransitives were observed, it would support the prevailing assumption that syntactic similarities between unaccusatives and passives trigger overpassivization errors with unaccusative verbs. Unfortunately, the results of Experiment 1 did not provide conclusive evidence for it. Thus, it remains uncertain whether Korean learners have the syntactic knowledge that passives and unaccusatives undergo a similar syntactic derivation (local A-movement), resulting in predominant overpassivization errors with unaccusatives. However, the results that Korean learners of English did not show native-like responses suggest that the syntax-prosody interface domain is an area where English learners hardly ever acquire a native-like understanding.

Examining the responses from the five most native-like learners reveals residual optionality, supporting the Interface Hypothesis, although further confirmation with a larger sample of highly advanced learners is needed. In addition, their native-like responses with passives and non-native-like responses with intransitives suggest that even highly advanced learners may not have fully acquired the two distinct syntactic derivations for intransitives. However, the fundamental reason for the overall non-native-like responses across learner groups remains unresolved. A clearer understanding of the mechanisms involved in acquiring the syntax–prosody interface may eventually shed light on this issue, specifically, whether the non-native-like responses stem from incomplete acquisition of the syntax-prosody interface or from the lack of acquisition of distinct syntactic derivations for each type of intransitive.^14^

To explore an alternative explanation, Experiment 2 was conducted to test whether overpassivization errors could arise from a simple analogy based on the thematic role (theme or patient) of subjects. Korean learners judged the agentivity of unaccusative subjects to be lower than that of unergative subjects, suggesting that the agentivity of the subject is pertinent to overpassivization errors with unaccusatives but not with unergatives. Given that agentivity is distinct from animacy, the results of this study further suggest that agentivity, independent of animacy, could contribute to overpassivization errors. In addition, lacking conclusive evidence to support a syntactic explanation, the account based on the semantic similarity between passives and unaccusatives could offer a more convincing perspective. Thus, the two experiments presented in this study suggest a reconsideration of the traditional syntactic account independent of entangled semantic properties. Otherwise, it remains unclear whether syntactic similarities truly play a role in prevalent overpassivization errors with unaccusative verbs.

Importantly, unlike prior studies that examined how non-syntactic factors influence the frequency of overpassivization, our study shifts the focus from error rates to the underlying representations learners employ. Although the present findings do not provide conclusive evidence that Korean L2 learners differentiate unaccusatives from unergatives on the basis of syntactic derivation, they do indicate that learners distinguish the thematic properties of subjects in terms of agentivity. This representational perspective allows us to reinterpret previously observed error patterns not as outcomes of syntactic misanalysis, but as consequences of how learners encode the semantic relations between subjects and predicates.

## Appendix

See Figs. 5, 6, 7 and 8.

## Footnotes

1 Legate (2003) argues that the application of the NSR provides evidence for the presence of phases on the VP of unaccusatives. Her proposal is rooted in Chomsky’s (2000) Phase theory, which posits that after constructing a phase (*v*P or CP), the complement of the phase head (VP or TP, respectively) is spelled-out to the interface levels (PF and LF). Legate assumes that if the spell-out domain contains more than one copy of a phrase, PF operation deletes every copy except the initial one. This assumption works as follows. Following Bresnan (1972), Legate assumes that the NSR applies cyclically. To illustrate, *the proposal* in (ia) was base-generated as the object in the embedded clause and then moved to its surface position, crossing the phase boundary. Consequently, the input to PF after the first phase formation is [left the proposal], in which just one copy of *the proposal* occurs. Thus, the deleting operation at PF does not apply to the noun, and the NSR applies to the final element, *the proposal*. After the last phase formation, the sentence stress assigned to the copy is realized with the highest copy which is overtly pronounced. This is why the non-final element bears the primary accent. In contrast, in (ib), the moved object *the dishes* does not bear sentence stress because the movement of the object occurs within a phase. The movement of *the dishes* occurs within a *v*P phase, and at the first spell-out, the spell-out domain contains two copies of *the dishes* as in [put *the dishes* away *the dishes*]. Thus, the PF operation that deletes non-initial copies deletes the last copy, and the input to the PF becomes [put the dishes away], with the NSR assigning sentence stress to the last element, *away*. Appying this analysis to unaccusatives predicts that if they have a phase at the VP level, the subject, base-generated as the complement of V, moves across the phase to the SpecTP. Since the first spell-out domain VP contains only one copy of the subject NP, the NSR assigns sentence stress to the subject, resulting in the subject-accent pattern. This prediction is borne out as we discussed in the current section.

(i) a. Mary liked the PROPOSAL that George left. (Bresnan. 1972:75, cited in Legate 2003: (9))
  b. Please put them/?the dishes AWAY. (Legate 2003: (10)).

2 It has been reported in the literature that Korean speakers can distinguish the two types of intransitives in their native language, Korean, as well (Allman 2016; Yang 1991). Diagnostics suggested for use in Korean include the obligatoriness of the nominative case marker on floating quantifiers or the availability of case alternation (see the references above). However, from our perspective, these observations are at times not sufficiently precise, as indicated in the corpus study by Allman (2016) regarding the case-marking of floating quantifiers. Additionally, these diagnostics are not associated with the surface word order between the verb and the subject, unlike their Spanish or Italian counterparts. Since distinguishing between the two types of intransitives does not necessarily imply that Korean speakers know the syntactic derivation of unaccusatives, we suggest that there is a lack of convincing evidence to make a conclusive remark.

3 Oshita’s observation (1997) aligns with the Full Transfer/Full Access (FT/FA) model of second language acquisition (Schwartz & Sprouse 1996), suggesting that the difference in the starting point of the L2 acquisition of English between Spanish/Italian speakers and Korean/Japanese speakers, which arguably reflects their first language grammar according to the FT/FA model, influences the pattern of L2 English production. Spanish learners of English, benefiting from an L1 transfer effect, support the V-subject NP order with unaccusative verbs, even though the target input may not include such a pattern (unless there is an expletive subject (e.g., *There arrived a strange package*). The issue of whether L2 learners categorize structures with an expletive subject as intransitive remains uncertain, and we leave this issue open for future studies, while concurrently delineating the scope of intransitives to sentences consisting solely of a verb and a subject NP). In contrast, for Korean learners of English, as the verb follows the subject in the basic word order (SOV) in Korean, neither L1 transfer effect nor the target language would support VS order with unaccusative verbs. We thank the anonymous reviewers for bringing up this point.

4 Additionally, syntax-based diagnostics cannot control the type of prosody participants have in their mind when making judgments. As discussed by Kitagawa and Fodor (2006), different prosodies can influence syntactic judgments. Therefore, the results of syntactic judgment tasks may not transparently reflect participants’ syntactic knowledge. We thank an anonymous reviewer for bringing this point to our attention.

5 Park and Usha (2007) observed that Korean L2 learners reject (incorrect) resultatives with unergative verbs but accept (correct) resultatives with alternating unaccusatives. They argued that these results show that Korean L2 learners know that unaccusatives have a single argument base-generated within VP, akin to a transitive object. While their observations are noteworthy to mention, the results do not explicitly reveal how Korean L2 English speakers understand the syntax of non-alternating unaccusatives. Given that overpassivization errors are frequently observed with non-alternating verbs, it might be premature to conclude that the results support the idea that syntactic similarity between passives and unaccusatives is the primary cause of overpassivization errors among Korean L2 English learners.

6 Ackema and Schoorlemmer (1995) show that middle constructions in Dutch, which select cognates of ‘have’, exhibit a pattern similar to unergatives rather than unaccusatives. If the subject is generated within VP and undergoes movement like a passive/unaccusative subject, middles would be expected to select cognates of ‘be’. Drawing from this observation, they argue that the subject of middles is an external NP.

7 We thank anonymous reviewers for bringing up this issue.

8 Due to sentence-final lengthening, the stressed syllable of the verb is consistently longer than the stressed syllable of the subject. While the duration of the subject remains constant across accent conditions, the verb is pronounced for a longer duration in the verb-accent condition compared to the subject-accent condition. In addition, the relative difference between the length of the stressed syllable of the verb and that of the stressed syllable of the subject in the verb-accent condition is much bigger than the difference observed in the subject-accent condition. Similar patterns are evident in all verb-type conditions.

9 An anonymous reviewer raised concerns about the interpretation of middle scores in a 5-point Likert scale, as discussed in Ionin & Zyzik (2014). We agree with the idea that participants could give the middle number 3 when they are not sure whether a given stimulus is natural/acceptable or unnatural/inacceptable. While we did not observe a noticeable pattern regarding the middle point in our data, we acknowledge the potential shortcomings of the methodology.

10 We thank anonymous reviewers for suggesting individual analyses.

11 We will not delve into the exact mechanism of stress assignment in different interpretations. For a detailed discussion on how the phase-based theory can be adapted to accommodate the subject-stress pattern in unergatives with the thetic interpretation, refer to Kratzer and Selkirk (2007).

12 We included the directed motion verb *come* in the unaccusative condition to ensure maximum equivalence with Experiment 1. We acknowledge that the subject of this verb can pass tests of agentivity when coupled with continuations like *on purpose*. However, in Experiment 2, we deliberately omitted such continuations, allowing participants to interpret experimental sentences with or without additional phrases. If participants perceive the subjects of unergatives as more agentive-like than those of unaccusatives even with this freedom of interpretation, we can interpret the results as indicating that L2 learners distinguish thematic roles assigned to subjects in the two types of intransitives. We appreciate the insight from an anonymous reviewer for highlighting this consideration.

13 We thank an anonymous reviewer for pointing this out.

14 We thank an anonymous reviewer for providing insightful comments.
